# *Brassica rapa* CURLY LEAF is a major H3K27 methyltransferase regulating flowering time

**DOI:** 10.1007/s00425-024-04454-7

**Published:** 2024-06-12

**Authors:** Laura Poza-Viejo, Miriam Payá-Milans, Mark D. Wilkinson, Manuel Piñeiro, José A. Jarillo, Pedro Crevillén

**Affiliations:** 1 Centro de Biotecnología y Genómica de Plantas (CBGP), Universidad Politécnica de Madrid (UPM) - Instituto Nacional de Investigación y Tecnología Agraria y Alimentaria (INIA/CSIC), Pozuelo de Alarcón, Madrid Spain; 2 Present Address: Centro de Investigación Biomédica en Red de Enfermedades Raras (CIBERER), FPS, Hospital Virgen del Rocío, Seville, Spain

**Keywords:** CURLY LEAF, *Brassica rapa*, Histone H3 methyltransferase, H3K27me3, Oilseed crop, Flowering time

## Abstract

**Main conclusion:**

In *Brassica rapa*, the epigenetic modifier BraA.CLF orchestrates flowering by modulating H3K27me3 levels at the floral integrator genes *FT*, *SOC1*, and *SEP3*, thereby influencing their expression.

**Abstract:**

CURLY LEAF (CLF) is the catalytic subunit of the plant Polycomb Repressive Complex 2 that mediates the trimethylation of histone H3 lysine 27 (H3K27me3), an epigenetic modification that leads to gene silencing. While the function of CURLY LEAF (CLF) has been extensively studied in *Arabidopsis thaliana*, its role in Brassica crops is barely known. In this study, we focused on the *Brassica rapa* homolog of *CLF* and found that the loss-of-function mutant *braA.clf-1* exhibits an accelerated flowering together with pleiotropic phenotypic alterations compared to wild-type plants. In addition, we carried out transcriptomic and H3K27me3 genome-wide analyses to identify the genes regulated by BraA.CLF. Interestingly, we observed that several floral regulatory genes, including the *B. rapa* homologs of *FT*, *SOC1* and *SEP3*, show reduced H3K27me3 levels and increased transcript levels compared to wild-type plants, suggesting that they are direct targets of BraA.CLF and key players in regulating flowering time in this crop. In addition, the results obtained will enhance our understanding of the epigenetic mechanisms regulating key developmental traits and will aid to increase crop yield by engineering new Brassica varieties with different flowering time requirements.

**Supplementary Information:**

The online version contains supplementary material available at 10.1007/s00425-024-04454-7.

## Introduction

Plants are complex organisms with intricate developmental processes that evolve in coordination with the surrounding environment. Within this context, the epigenetic mechanisms regulating gene expression play a pivotal role in orchestrating growth, development and differentiation (Schuettengruber et al. 2017; Baile et al. 2022). Polycomb Group (PcG) complexes are epigenetic regulators that transcriptionally repress target genes through the deposition of posttranslational histone modifications (Schuettengruber et al. 2017). The Polycomb Repressive Complex 2 (PRC2) catalyzes the trimethylation of histone H3 lysine 27 (H3K27me3), which eventually leads to the silencing of gene expression (Guo et al. 2021). PRC2 was initially discovered in Drosophila where it was shown to contain four core subunits: Enhancer of Zeste (E(z)), Extra sex combs (Esc), Suppressor of Zeste 12 (Su(z)12), and Nucleosome remodelling factor 55 (Nurf55) (Schuettengruber et al. 2017). In *Arabidopsis thaliana*, extensive studies have found that PRC2 subunits are conserved although they are encoded by multiple genes: CURLY LEAF (CLF), SWINGER (SWN), and MEDEA (MEA) are homologs of E(z); VERNALIZATION 2 (VRN2), EMBRYONIC FLOWERING 2 (EMF2) and FERTILIZATION-INDEPENDENT SEED 2 (FIS2) are homologs of Su(z)12; FERTILIZATION-INDEPENDENT ENDOSPERM 1 (FIE1) is the homolog of ESC; and there are five MULTI-SUBUNIT SUPPRESSOR OF IRA proteins (MSI1–5) homologs of *Nurf55* (Vijayanathan et al. 2022; Godwin and Farrona 2022).

The *A. thaliana* PRC2 E(z)-like catalytic subunits CLF, SWN and MEA have different expression profiles and specialized functions (Kinoshita et al. 1999; Chanvivattana et al. 2004). The *MEA* gene exhibits imprinting and it is expressed in the female gametophyte and endosperm, being required to silence the paternal *MEA* allele after fertilization (Grossniklaus et al. 1998; Kinoshita et al. 1999). CLF acts as the primary H3K27 methyltransferase in the sporophyte, and is partially redundant with SWN (Chanvivattana et al. 2004; Shu et al. 2020). Mutations in *CLF* lead to pleiotropic developmental defects, whereas *swn* mutants exhibit subtle developmental abnormalities (Goodrich et al. 1997; Chanvivattana et al. 2004). Notably, *SWN* mutations considerably enhance *clf* phenotypic alterations, and the *clf swn* double mutant displays callus-like structures and somatic embryos (Chanvivattana et al. 2004).

The strong developmental defects displayed by *clf* mutants include small plant size, curved leaves and accelerated flowering time. The leaf curling phenotype is caused by the misregulation of the floral homeotic genes *AGAMOUS* (*AG*) and *SEPALLATA3* (*SEP3*) (Goodrich et al. 1997; Lopez-Vernaza et al. 2012). Meanwhile, the early-flowering phenotype is at least partially due to the upregulation of the floral integrator gene *FLOWERING LOCUS T* (*FT*) (Farrona et al. 2011). A number of studies have also highlighted the critical role of CLF in the regulation of cell proliferation and meristematic activity (Shu et al. 2020). For example, CLF represses the expression of differentiation-promoting genes in the shoot apical meristem, thereby sustaining the undifferentiated state of root and floral stem cells (Liu et al. 2011). However, the function of CLF extends beyond developmental processes and encompasses plant immunity and lipid metabolism (Liu et al. 2016; Singkaravanit‐Ogawa et al. 2021). In fact, genome-wide analyses show direct binding of CLF protein to hundreds of *A. thaliana* target genes involved in a number of biologic processes (Wang et al. 2016; Shu et al. 2019).

The Brassica genus comprises a variety of vegetables, condiments, and economically significant oilseed crops that are closely related to *A. thaliana*. Within the Brassica genus, *Brassica rapa* holds economic significance globally. This species displays remarkable variations in morphology resulting in different agricultural uses including leafy vegetables like Chinese cabbage, enlarged roots in turnip, and oilseed varieties such as yellow sarson. The genome of *B. rapa* is fully sequenced and contributes to half of the genomes of the allotetraploid crops *B. juncea* (Indian mustard) and *B. napus* (oilseed rape) (Wang et al. 2011; Zhang et al. 2018). Recent advances in *B. rapa* epigenomics have provided valuable insights into the regulatory mechanisms governing gene expression in this important crop species. For example, the repressive histone modification H3K27me3 has been proposed to play a key role in the regulation of the floral transition (Payá-Milans et al. 2019; Poza-Viejo et al. 2022). Furthermore, there is a distinct distribution of H3K36me3 and H3K27me3 among homoeologous paired genes, which has been proposed to lead to variations in gene-expression levels or tissue specificity (Mehraj et al. 2021).

Despite the significant progress made in understanding the function of PRC2 in *A. thaliana,* the role of this gene in Brassica crops is only beginning to be understood. We previously isolated *braA.clf-1,* a mutant line in *B. rapa* R-o-18 with abnormalities that resemble some of the classic *A. thaliana clf*-mutant phenotypes (Payá-Milans et al. 2019). One of the most conspicuous *A. thaliana clf* phenotypic alterations is an early flowering. However, the flowering time of *braA.clf-1* has not been described yet. Here, we report that *braA.clf-1* shows an acceleration of flowering time in comparison to wild-type plants. In addition, to unveil the underlying genomic defects, we performed transcriptomics and genome-wide analysis of H3K27me3 in *braA.clf-1* leaves. To our knowledge, this is the first genome-wide study of an epigenetic mark of a Polycomb mutant in a Brassica crop. Our data show that a number of floral integrator genes, including the *B. rapa* homologs of *FT* and *SUPPRESSOR OF OVEREXPRESSION OF CO 1* (*SOC1*), display reduced H3K27me3 occupancy and higher transcript levels in *braA.clf-1* that are consistent with its early-flowering phenotype.

## Materials and methods

### Plant materials and growth conditions

We worked with *B. rapa* R-o-18, an inbred variety of *Brassica rapa subsp. trilocularis* (Yellow Sarson) that has been widely studied as a model oilseed crop (Stephenson et al. 2010). The TILLING (Targeting Induced Local Lesions In Genomes) mutant line *braA.clf-1* (JI32391-A) was obtained from RevGenUK. Plants were grown in controlled-environment growth chambers under long day conditions of 16 h of light with day/night temperatures of 21/19 °C and mix of cool-white and wide-spectrum FLOURA fluorescent lights (100 µE/m^2^s).

### Flowering time analysis

*B. rapa* flowering time experiments were performed in controlled-environment chambers using 12 cm diameter pots. Flowering time was quantified using three metrics: the number of days from germination to bolting, the number of days until the first flower opened, and the number of leaves present at bolting.

### Chromatin immunoprecipitation and sequencing

The *braA.clf-1* plants used in our genomic experiments were grown in parallel and collected together with the plant materials described in Poza-Viejo et al. 2022. We used primary leaves of *B. rapa* plants grown for 14 days collected at the end of the light period (Zeitgeber time ZT16).

Chromatin immunoprecipitation (ChIP) followed by high-throughput sequencing (ChIP-seq) experiments were performed using an anti-H3K27me3 antibody (Diagenode C15410195) as described in Poza-Viejo et al. (2019). Two biologic ChIP replicates (one leaf from 8 independent plants) were processed (Table S1). Input contained a pool of DNA from all genotypes and it was sequenced at greater depth to improve peak-calling identification. ChIP-seq libraries were prepared using NEBNext Ultra DNA Library Prep kit (New England BioLabs) and sequenced at 2 × 50 bp paired-end reads by the Genomics Unit of the Centro Nacional de Análisis Genómico CNAG-CRG (Barcelona, Spain).

For the ChIP-qPCR assays, DNA was amplified using real-time quantitative PCR (qPCR) with the primers listed in Table S2. The enrichment of ChIP DNA was calculated as the percentage of immunoprecipitated DNA relative to the input DNA normalized to the concentration of total DNA content determined by QUBIT fluorometer (ThermoFisher Scientific).

### Transcriptomic and gene-expression analyses

Transcriptome analysis was performed by RNA sequencing (RNA-seq). Total RNA was extracted from the same plants used for the ChIP-seq using E.Z.N.A. Plant RNA Kit (Omega Bio-tek). At least three biologic replicates (one leaf from eight independent plants) were processed for each genotype (Table S3). RNA-seq libraries were prepared and sequenced at 2 × 100 bp paired-end reads by MACROGEN Inc. (Korea).

For reverse transcription quantitative PCR (RT-qPCR) expression analyses, RNA extraction and cDNA synthesis were performed using the E.Z.N.A. Plant RNA Kit (Omega Bio-tek) and the Maxima First Strand cDNA Synthesis Kit (ThermoFisher Scientific), respectively, following the manufacturers’ guidelines. The RT-qPCR data were presented as relative mRNA levels, calculated using the 2^−ΔΔCT^ method, with *BraA.TUBULIN* (*BraA10g026070.3C*) serving as the housekeeping gene (Xu et al. 2014). The primers used for RT-qPCR can be found in Table S2.

### ChIP-seq and RNA-seq computational analyses

Transcriptomic and epigenomic data were analyzed using our previously described analytical workflow (Payá-Milans et al. 2019; Poza-Viejo et al. 2022) at the Supercomputing Galician Centre (CESGA) high-throughput computing server. The specific code can be found at https://github.com/mpaya/epigenomics_scripts. Briefly, raw reads were trimmed with Skewer v0.2.2 (Jiang et al. 2014), then mapped to the *B. rapa* Chiifu v3.0 genome with the fast aligner Bowtie2 v2.3.5 (Langmead and Salzberg 2012), and mapping metrics were collected with Picard v2.21.1 (2018). For RNA-seq samples, counts were obtained with htseq-count v0.11.2 (Anders et al. 2015) and differentially expressed genes (DEG) were determined with DESeq2 (Love et al. 2014). The correlation of gene-expression profiles between RNA-seq samples is shown in Fig. S1. Analysis of ChIP-seq data was performed using Bowtie2 v2.3.5 as read aligner, Epic2 (Stovner and Sætrom 2019) as peak caller, and a quantitative comparison of mutant vs wild-type ChIP-seq signal was performed with MAnorm v1.2.0 (Shao et al. 2012). Peaks were annotated to overlapping genes of *B. rapa* at 500 bp distance using ChIPpeakAnno R package (Zhu et al. 2010). ChIP-seq replicates yielded consistent results (Fig. S2).

### Other bioinformatic analyses

Custom annotation of gene models was obtained by comparing *B. rapa* genome v3.0 coding sequences against Arabidopsis (TAIR10 proteins, blastx of *B. rapa* coding sequences with an E-value cutoff of 1e − 25) using BLAST (Basic Local Alignment Search Tool). The phylogenetic tree was obtained using the web service Phylogeny.fr (http://www.phylogeny.fr/index.cgi) (Dereeper et al. 2008). Sequences were aligned using MUSCLE, and ambiguous regions were eliminated with Gblocks post-alignment; the PhyML program was utilized for maximum likelihood-based reconstruction of the phylogenetic tree, and TreeDyn program was employed for graphical representation (Dereeper et al. 2008). Singular Enrichment Analysis (SEA) of Gene Ontology (GO) terms analyses were conducted utilizing agriGO v2.0 employing Fisher statistical test approach, Yekutieli Multi-test adjustment technique (*p* ≤ 0.05), and Plant GO slim ontology category (Du et al. 2010). To reduce the complexity and redundant GO terms we used REVIGO (Supek et al. 2011) with default parameters (allowed similarity = 0.7; semantic similarity measure = SimRel). Graphs and statistical analyses were performed using GraphPad Prism 9 (www.graphpad.com). Venn diagrams were drafted using the free web tool DeepVenn (Hulsen 2022). The hypergeometric test was calculated using the Hypergeometric *P* value calculator from the Graeber Lab (https://systems.crump.ucla.edu/hypergeometric/). The Integrative Genomic Viewer (IGV) software (Thorvaldsdottir et al. 2013) was used to visualize H3K27me3 ChIP-seq peaks.

## Results

### CURLY LEAF is a single-copy gene in *Brassica* rapa

**Fig. 1:**
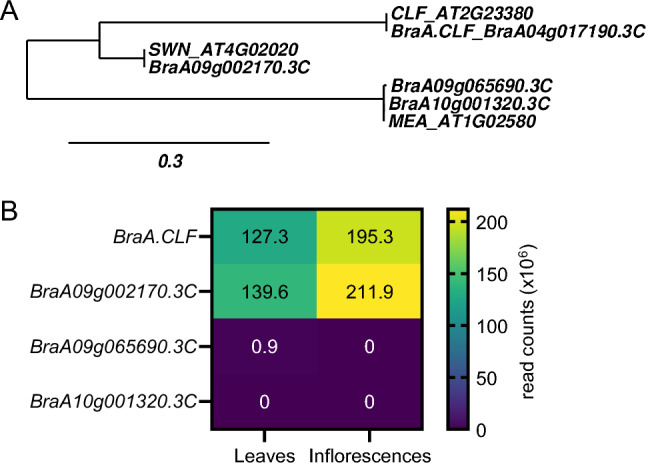
Homologs of the *A. thaliana* H3K27me3 methyltransferases in *B. rapa.* **A** Phylogenetic tree showing the relationship between *A. thaliana* H3K27me3 methyltransferases (CLF AT2G23380, SWN AT4G02020 and MEA AT1G02580) and its *B. rapa* homologs (BraA.CLF BraA04g017190.3C, SWN homolog BraA09g002170.3C and MEA homologs BraA10g001320.3C and BraA09g065690.3C). **B** Number of transcripts (cpm, counts per million) of *B. rapa* H3K27me3 methyltransferases-encoding genes found in leaves and inflorescences. Data obtained from Payá-Milan et al*.* 2019

To investigate the functional role of H3K27me3 methyltransferases in Brassica crops, we looked for homologs of the *A. thaliana* CLF (AT2G23380) protein using BLAST and performed a phylogenetic analysis. Consistent with the findings reported by Huang et al. (2011) we determined that BraA.CLF (BraA04g017190.3C) is the sole CLF homolog, and identified one homolog to SWN (BraA09g002170.3C) as well as two homologs for MEA (BraA09g065690.3C and BraA10g001320.3C) within the updated *B. rapa* genome assembly V3.0 (Zhang et al. 2018) (Fig. 1A). Our analysis of the most recent *B. rapa* genome assembly V4.0 yielded similar results (Zhang et al. 2023).

In *A. thaliana,* the genes encoding the three histone H3K27me3 methyltransferases are differentially expressed. *CLF* and *MEA* genes are expressed in a variety of tissues, with *MEA* expression being highest in the endosperm (Kinoshita et al. 1999; Chanvivattana et al. 2004; Shu et al. 2020). We studied the expression of the four *B. rapa* H3K27 methyltransferase genes using our published RNA-seq data (Payá-Milans et al. 2019). We found that *BraA.CLF* and *BraA.SWN* genes were expressed in *B. rapa* R-o-18 leaves and inflorescences, whereas the expression of *B. rapa MEA* homologs was not detected in these experiments (Fig. 1B). We then investigated *B. rapa MEA* homologs in public transcriptomic databases at the Brassicaceae Database (BRAD) (Cheng et al. 2011) and found that *BraA10g001320.3C* was not detected in any tissue or growth condition, while *BraA09g065690.3C* was only expressed in siliques. This suggested that *B. rapa MEA* gene functions would be restricted to the gametophyte, whereas *CLF* and *SWN* may have a prominent role during the sporophyte phase. Although further research is needed, all these data are consistent with a conserved expression pattern of the H3K27 methyltransferases-encoding genes in *B. rapa* and *A. thaliana*.

### BraA.CLF contributes to the repression of the floral transition

**Fig. 2:**
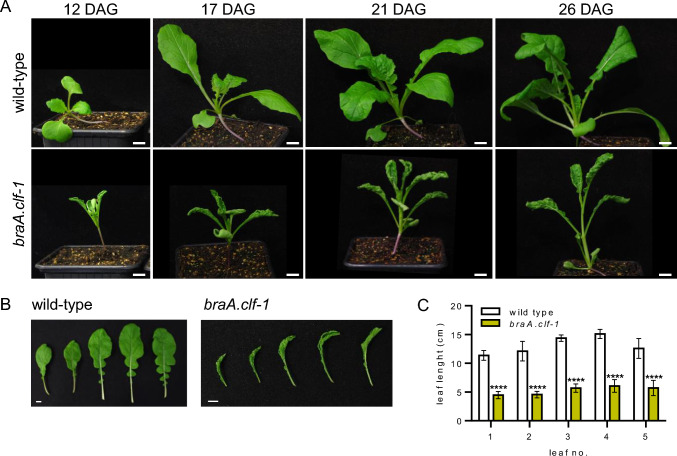
Phenotypic alterations of *braA.clf-1*. **A** Picture of *braA.clf-1* and wild-type plants at 12-, 17-, 21- and 26-days after germination (DAG); scale bar = 1 cm. **B** Picture showing severe abnormalities in the leaf shape and size of *braA.clf-1* compared to wild-type plants at 26 DAG; scale bar = 1 cm. **C** Quantification of leaf length measured from the tip to the end of the petiole of the first five leaves of *braA.clf-1* and wild-type plants. Mean values ± SD (*n* = 10). Statistical significance was calculated using one-way analysis of variance (ANOVA) followed by Sidak’s multiple comparison test (****P value < 0.0001)

**Fig. 3:**
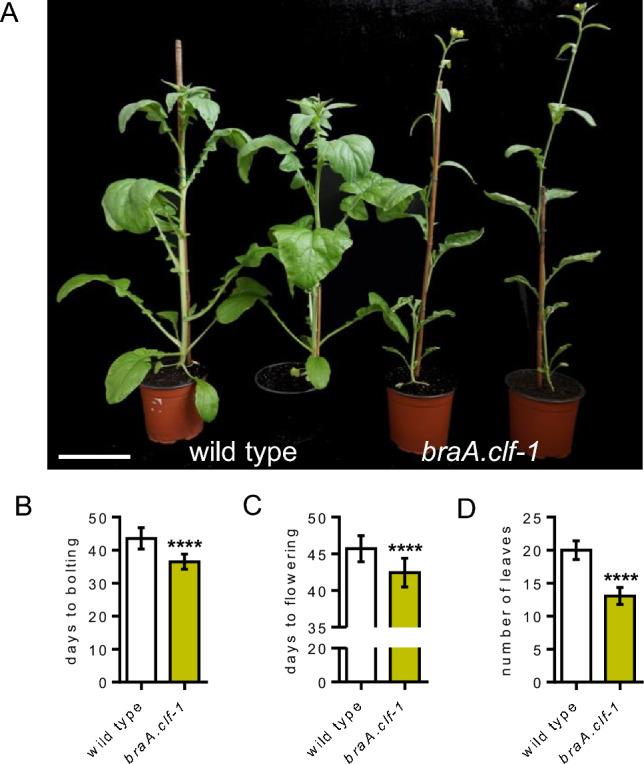
BraA.CLF acts as a floral repressor. **A** Picture of *braA.clf-1* and wild-type plants at the flowering stage; scale bar = 13 cm. **B**–**D** Flowering time of *braA.clf-1* and wild-type plants was measured as days from germination to bolting (**B**), days from germination to the opening of the first flower (**C**), and the number of primary leaves generated by the plant until the first flower opens (**D**). Mean values ± SD (*n* = 15). Statistical significance was calculated using the Student´s t-test (****P value < 0.0001)

We previously isolated *braA.clf-1* (Payá-Milans et al. 2019), a TILLING mutant line that carries a stop-codon (Q615*) upstream of the CXC and catalytic SET domain in the *BraA.CLF* gene (Fig. S3), although its flowering time phenotype was not evaluated at that time. Prior to the flowering phenotypic analysis, the mutant *braA.clf-1* was backcrossed twice to the wild-type parental line to reduce the load of secondary TILLING mutations. Homozygous plants carrying the *braA.clf-1* mutation derived from these backcrosses exhibited pleiotropic phenotypic alterations as previously observed (Payá-Milans et al. 2019). For instance, the stem of *braA.clf-1* plants was thinner than that of the wild-type plants (Figs. 2 and 3A). The leaf length and size was also altered in the mutant (Fig. 2B) with the first five leaves of *braA.clf-1* mutants displaying on average a 50% length reduction compared to the wild-type leaves (Fig. 2C). However, we could not observe any abnormally elongated pistil or severe reduction in plant height as described by Nugroho et al. (2023). In our study, both *braA.clf-1* and wild-type plants attained similar heights at the flowering stage (Fig. 3A). These discrepancies may be attributed either to different growth conditions, or the use of backcrossed mutant lines in our study.

In *A. thaliana,* the expression of many floral regulatory genes is controlled by H3K27me3 methylation (Baile et al. 2022; Vijayanathan et al. 2022). We have also observed a similar scenario in *B. rapa* (Payá-Milans et al. 2019; Poza-Viejo et al. 2022)*.* Thus, we decided to characterize the flowering time of the *braA.clf-1* mutant. For that, we grew plants in controlled-climate chambers and observed that *braA.clf-1* plants exhibited an early-flowering phenotype compared to wild-type *B. rapa* R-o-18 plants. (Fig. 3B–D). This flowering time acceleration was statistically significant when quantified as the number of days before reaching the bolting stage or the number of days prior to opening of the first flower (Fig. 3B–C). In addition, we also observed that the number of leaves developed by the *braA.clf-1* mutant before flowering, a commonly used parameter to estimate flowering time in *A. thaliana*, was reduced compared to wild-type plants (Fig. 3D). All these observations demonstrate that *braA.clf-1* is an early-flowering mutant, and suggest that BraA.CLF could be repressing the expression of key flowering promoting genes.

### The* braA.clf-1* mutant displays genome-wide alterations in H3K27me3 levels

**Fig. 4:**
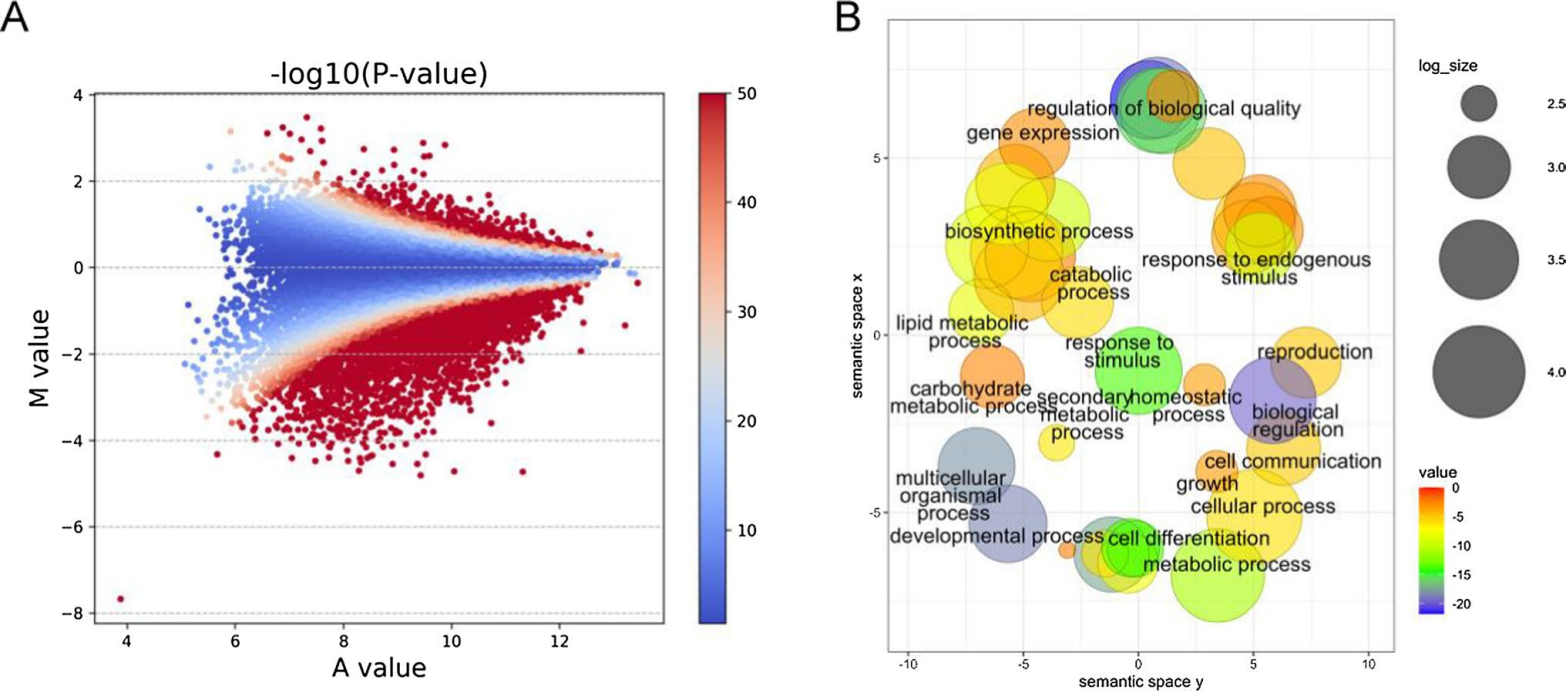
H3K27me3 epigenomic analysis of *braA.clf-1*.** A** MA plot showing the differential accumulation of H3K27me3 on ChIP‐seq peaks determined with MAnorm. Significance is indicated with color scale, red indicates − log10 (*P* value) > 50. Normalized read densities of *braA.clf-1* relative to the wild type were compared to represent the average signal strength of samples (A value) against their log2 fold‐change (M value) of each peak. **B** Semantic clustering of enriched GO terms of H3K27me3 hypomethylated genes in *braA.clf-1* mutant. SEA was performed with agriGO and the significant GO terms were clustered with the REVIGO tool. The size of the bubble indicates the frequency of the GO term and *P* values are indicated by a color scale

To investigate the functional role of BraA.CLF as a histone methyltransferase in *B. rapa*, we performed H3K27me3 ChIP-seq experiments on *braA.clf-1* mutants and wild-type leaves. Immunoprecipitated DNA samples were sequenced using high-throughput next-generation sequencing (see Methods and Supplementary Table S1). After the ChIP-seq bioinformatic analysis, we identified 7385 genes with altered levels of H3K27me3 in the *braA.clf-1* mutant compared to the wild type (Fig. 4A and Supplementary Data S1; |M value|≥ 0.5 and *P* value ≤ 0.1). Among these genes, 4219 were hypomethylated and represented likely targets of BraA.CLF methyltransferase activity. This number accounts for over 30% of the H3K27me3-marked genes in leaves of *B. rapa* (Payá-Milans et al. 2019). Remarkably, there was also an increase of H3K27me3 in a number of genes in *braA.clf-1*. This phenomenon has also been observed in previous studies in other plant and animal species, and it is likely due to an indirect effect (Wang et al. 2016).

To unravel the functional implications of BraA.CLF activity, we performed a SEA-GO analysis of the set of *braA.clf-1* hypomethylated genes using AgriGO (Supplementary Data S2). The GO term list obtained was condensed and represented using the REVIGO tool (Fig. 4B). We found that among the most overrepresented biological process GO categories were: regulation of gene expression (GO:0010468), biological regulation (GO:0065007) and regulation of metabolic process (GO:0019222). There were also several GO categories related to regulation of cellular process (GO:0050794) and response to stimulus (GO:0050896). Consistent with a key role of BraA.CLF repressing floral homeotic genes in leaves (Payá-Milans et al. 2019), we also found the GO category of developmental process (GO:0032502) including floral development (GO:0009908) enriched in the list of hypomethylated genes (Supplementary Data S2 and Fig. 4B).

**Fig. 5:**
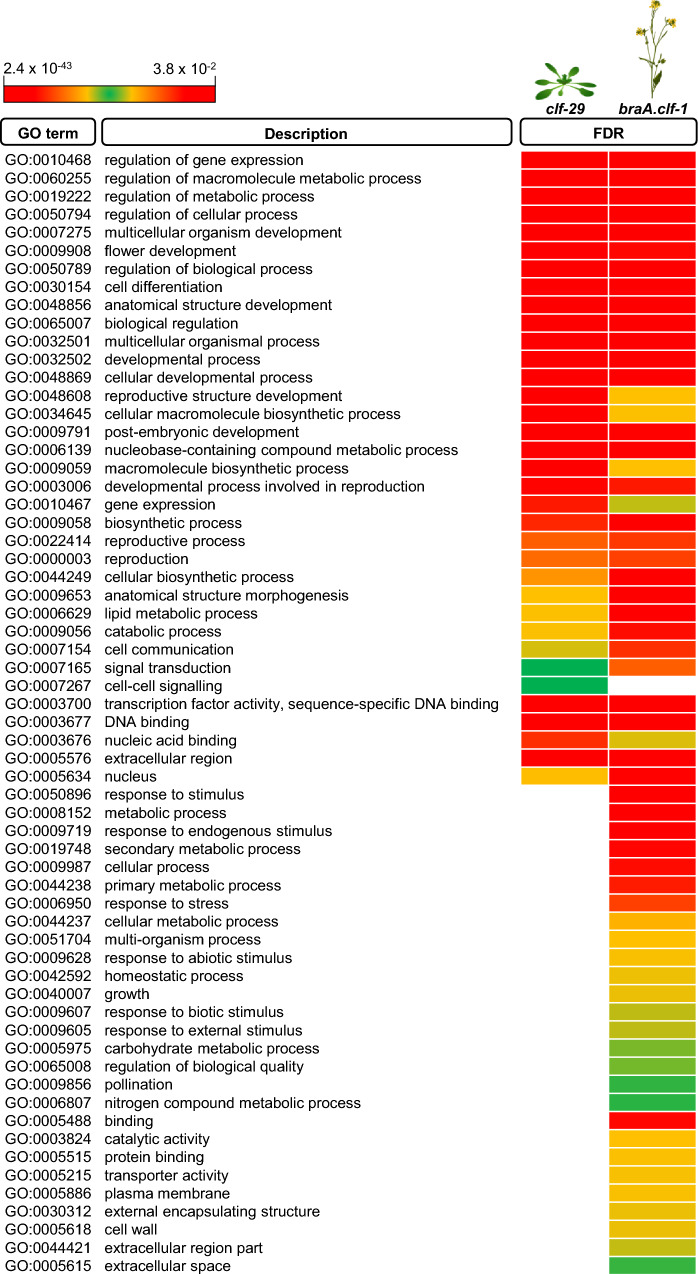
Comparison of enriched GO categories between hypomethylated genes from *braA.clf-1* and *A. thaliana clf-29*. Comparison between common and exclusive enriched GO terms from *A. thaliana clf-29* (Wang et al. 2016) and our *braA.clf-1* mutant datasets using the SEACOMPARE tool from AgriGO. The color indicates the FDR value

To compare our data with *A. thaliana clf* data sets available, we first identified homologs of our list of hypomethylated genes in *B. rapa* using BLAST, and removed any duplicated *A. thaliana* gene terms. Next, we compared the set of hypomethylated and hypermethylated genes in *braA.clf-1* with different *clf* datasets in *A. thaliana*, which varied in the number of identified hypomethylated genes (Wang et al. 2016; Carter et al. 2018; Shu et al. 2019). We found a statistically significant overlap in all cases (Fig. S4). We then performed a comparative enrichment analysis of GO terms between the datasets of hypomethylated from Wang et al. (2016) and our *braA.clf-1* mutant using the SEACOMPARE tool from AgriGO. Despite comparing data from different sample materials (2 weeks old *A. thaliana* plants *vs B. rapa* leaves) and experiments performed in different laboratories, we found an extensive coincidence between the enriched GO terms categories between hypomethylated genes in *B. rapa braA.clf-1* and *A. thaliana clf* (Fig. 5). These data suggest that *BraA.CLF*, as a key component of the PRC2 complex, plays a role in the regulation of several biological processes that are conserved between *B. rapa* and *A. thaliana*.

### The* braA.clf-1* mutant exhibits a large number of misregulated genes

**Fig. 6:**
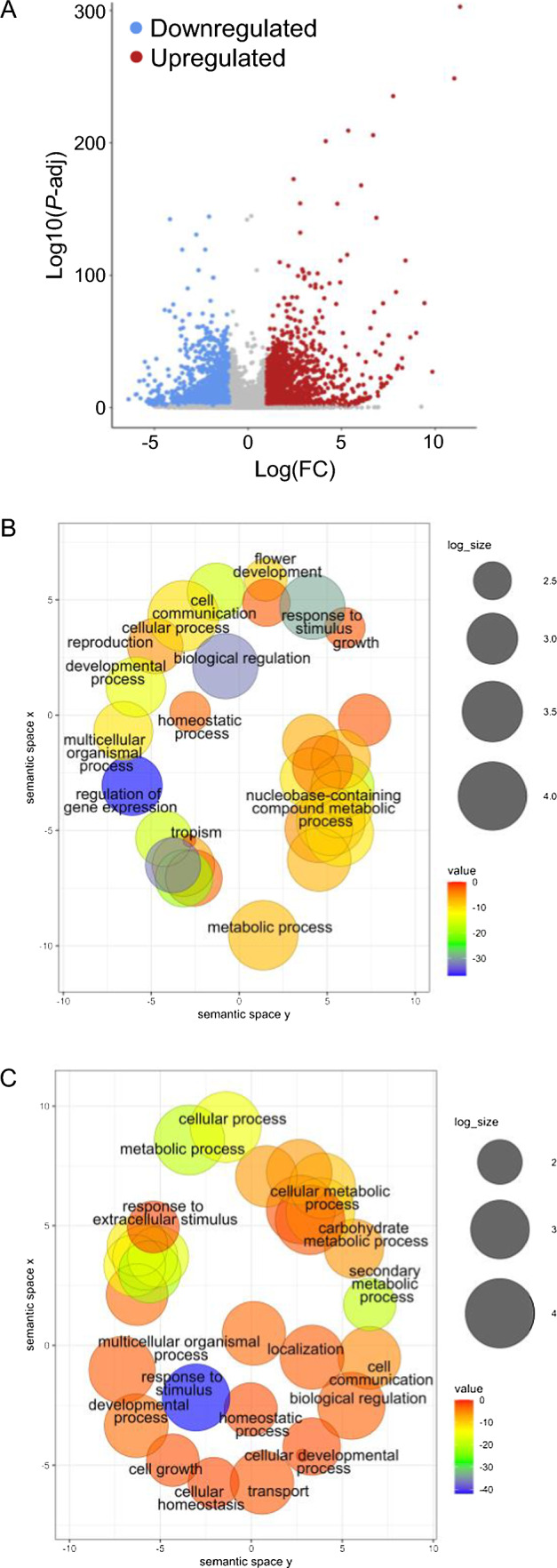
Transcriptomic analysis of the *braA.clf-1.* **A** Volcano plot showing the differentially expressed genes in *braA.clf‐1* compared to wild-type plants. Upregulated genes (red) and downregulated genes (blue) are shown (DESeq2 |Log_2_(FC)|≥ 1; *P*-adj ≤ 0.1). **B**–**C** SEA-GO analysis of upregulated (**B**) and downregulated (**C**) genes in *braA.clf-1*. Data were represented using REVIGO to reduce complexity

In combination with our ChIP-seq, we performed a transcriptomic study by RNA-seq of *braA.clf-1* mutant and wild-type leaves (see Methods and Supplementary Table S3 for details). Following the bioinformatic analysis, we identified 2159 genes that were upregulated, and 2116 genes that were downregulated in comparison to the wild type. (Fig. 6A and Supplementary Data S3; DESeq2 |log2(FC)|≥ 1; *P*-adj ≤ 0.1). This large number of misregulated genes was consistent with the pleiotropic phenotype of *braA.clf-1* mutant, which may be due to direct and indirect effects of the methyltransferase activity of CLF in *B. rapa*.

To gain insight into the biological processes affected in the mutant, we then performed a SEA-GO analysis of the *braA.clf-1* upregulated and downregulated set of genes (Fig. 6B and C, and Supplementary Data S4 and S5). The top-enriched Gene Ontology (GO) categories in the set of upregulated genes in *braA.clf-1* included regulation of biological processes (GO:0050789), regulation of gene expression (GO:0010468), and regulation of macromolecule metabolic processes (GO:0060255) (Supplementary Data S4). In addition, the list of upregulated genes included several GO terms related to cellular processes (GO:0050794), response to stimulus (GO:0050896), and developmental processes (GO:0032502), such as flower development (GO:0009908). The set of downregulated genes was enriched in several terms related to response to stimulus (GO:0050896 and GO:0009628), cellular processes (GO:0009987, GO:0044237, GO:0044249), and metabolism (GO:0008152 and GO:0019748) (Supplementary Data S5). Notably, we observed several GO categories related to the regulation of development or metabolism that were enriched in both gene sets.

To sum up, our transcriptomic analyses suggest that while BraA.CLF plays a prominent role in regulating developmental genes, it also directly or indirectly regulates a large number of genes related to other biological processes, including metabolism.

### BraA.CLF modulates the expression of a number of floral integrator genes

**Fig. 7:**
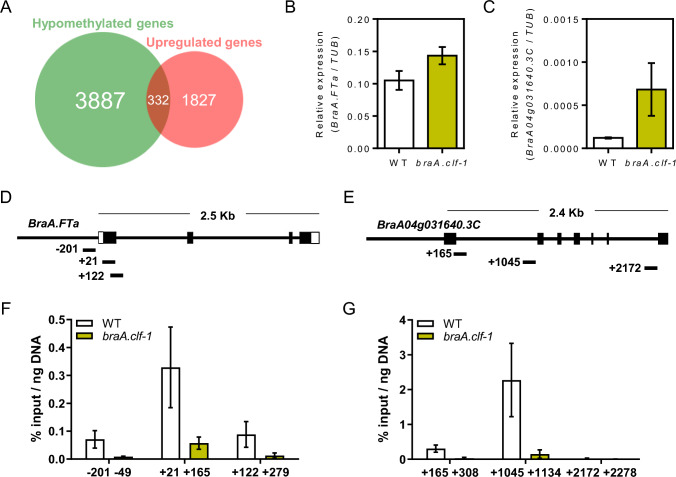
BraA.CLF modulates the expression of floral regulatory genes. **A** Venn diagram showing the overlap between hypomethylated and upregulated genes of *braA.clf-1* mutant. The overlap is over enriched 1.54 fold compared to expectations (hypergeometric test, *P* value = 2.21e-16). **B**–**C** RT-qPCR data showing the relative expression of *BraA.FTa* locus (**B**) and BraA04g031640.3C (*BraA.SOC1*) (**C**) in leaves of wild-type and *braA.clf-1* mutant plants. Bars represent the average of two replicates for each experiment *(n* = 2), and error bars represent the standard deviation error. Mean values ± SD (*n* = 2). **D**–**E** Representation of *BraA.FTa* (**D**) and *BraA.SOC1 (BraA04g031640.3C)* (**E**) loci showing the regions analyzed by ChIP-qPCR. **F**–**G** ChIP-qPCR data showing the H3K27me3 enrichment over *BraA.FTa* locus (**F**) and *BraA04g031640.3C (SOC1)* (**G**) in leaves of wild-type and *braA.clf-1* mutant plants. Mean values ± SD (*n* = 2)

To explain the accelerated flowering time of the *braA.clf-1* mutant, we compared the genes with reduced levels of H3K27me3 (hypomethylated) and increased expression (upregulated) in *braA.clf-1.* We identified a set of 332 genes (Fig. 7A), which likely includes the direct target genes of BraA.CLF contributing to the mutant phenotype. Then, we determined the *A. thaliana* homologs by BLAST analysis and performed a cross-comparison with the FLOweRing Interactive Database (FLOR-ID), a curated list of more than 306 floral regulatory genes in *A. thaliana* (Bouché et al. 2016). Following this reasoning, we discovered 23 *B. rapa* genes hypomethylated and upregulated in *braA.clf-1* directly associated with the floral transition (Table 1).

**Table 1:** Floral regulatory genes upregulated and H3K27-hypomethylated in *braA.clf-1*

*B. rapa*	*A. thaliana*	Log2(FC)	*M* value	Symbol	Description
BraA09g056690.3C	AT1G19330.1	1.34	− 0.62	AFR2	Histone deacetylase complex subunit
BraA07g029500.3C	AT1G71692.1	1.65	− 0.90	AGL12	MADS-box transcription factor
BraA09g054680.3C	AT2G22630.2	7.13	− 1.50	AGL17	MADS-box transcription factor
BraA03g050620.3C	AT4G22950.1	6.15	− 0.51	AGL19	MADS-box transcription factor
BraA03g051930.3C	AT4G24540.1	1.54	− 0.78	AGL24	MADS-box transcription factor
BraA06g025660.3C	AT5G62165.17	1.87	− 3.19	AGL42	MADS-box transcription factor
BraA02g043490.3C	AT5G62165.13	5.31	− 1.94	AGL42	MADS-box transcription factor
BraA03g015280.3C	AT5G51860.2	7.18	− 1.34	AGL72	MADS-box transcription factor
BraA01g016140.3C	AT5G51860.2	4.97	− 1.15	AGL72	MADS-box transcription factor
BraA02g039100.3C	AT3G30260.1	6.16	− 3.06	AGL79	MADS-box transcription factor
BraA06g036210.3C	AT3G30260.1	5.19	− 2.17	AGL79	MADS-box transcription factor
BraA01g034360.3C	AT3G18550.3	3.92	− 2.00	BRC1	TCP transcription factor
BraA03g038400.3C	AT3G18550.1	3.82	− 1.52	BRC1	TCP transcription factor
BraA02g016700.3C	AT1G65480.1	1.05	− 0.62	FT	PEBP-like protein/floral integrator gene
BraA03g002880.3C	AT5G07200.1	1.62	− 0.79	GA20OX3	Gibberellin 20-oxidase
BraA09g003630.3C	AT3G28910.1	1.30	− 3.58	MYB30	MYB transcription factor
BraA07g012310.3C	AT1G24260.2	6.31	− 3.82	SEP3	MADS-box transcription factor/homeotic gene
BraA09g037210.3C	AT1G24260.2	11.02	− 2.15	SEP3	MADS-box transcription factor/homeotic gene
BraA08g025030.3C	AT1G24260.2	7.90	− 1.13	SEP3	MADS-box transcription factor/homeotic gene
BraA04g031640.3C	AT2G45660.1	1.23	− 3.60	SOC1	MADS-box transcription factor/floral integrator gene
BraA03g023790.3C	AT2G45660.1	3.30	− 0.82	SOC1	MADS-box transcription factor/floral integrator gene
BraA05g005370.3C	AT2G45660.1	2.65	− 0.54	SOC1	MADS-box transcription factor/floral integrator gene
BraA09g036170.3C	AT1G28520.4	1.50	− 1.79	VOZ1	Zinc Finger transcription activator

The list was generated comparing the curated list of flowering regulators in *A. thaliana* described in the FLOweRing Interactive Database (FLOR-ID) (Bouché et al. 2016) with genes upregulated and H3K27me3-hypomethylated in *braA.clf-1*

The early-flowering phenotype observed in the *A. thaliana clf* mutant is caused by the upregulation of the floral integrator gene *FT* and the homeotic gene *SEP3* (Jiang et al. 2008; Lopez-Vernaza et al. 2012). We found several homologs of the AGAMOUS-like MADS-box transcription factor family that have been implicated in flowering time regulation in *A. thaliana*. Among them, we identified three *B. rapa SEP3* homologs that were hypomethylated and upregulated in *braA.clf-1* (Table 1). We also found the three homologs of *B. rapa SOC1* and the main *B. rapa FT*, *BraA.FT.a* or *FT1* (Table 1), to be hypomethylated and upregulated in the *braA.clf-1* mutant compared to the wild type. The increased mRNA expression levels and decreased H3K27me3 levels in *braA.clf-1* compared to the wild type for *BraA.FT.a* and *BraA04g031640.3C* (*BraA.SOC1*) were confirmed through independent RT-qPCR (Fig. 7B–C) and ChIP-qPCR experiments (Fig. 7D–G). All these floral integrator genes have been shown to promote flowering in *B. rapa* (del Olmo et al. 2019; Calderwood et al. 2021; Wang et al. 2022) and their upregulation is likely responsible for the early-flowering phenotype observed in the *braA.clf-1* mutant.

On the other hand, in *A. thaliana* CLF also regulates the floral repressor *FLOWERING LOCUS C* (*FLC*), but its effects on flowering time are masked by the increased *FT* expression in the *clf* mutant (Doyle and Amasino 2009; Lopez-Vernaza et al. 2012). However, there was no *B. rapa FLC-like* gene in our *braA.clf-1* ChIP-seq or RNA-seq datasets. Further research will be required to define whether BraA.CLF regulates the expression of *FLC-like* genes in other *B. rapa* cultivars.

## Discussion

Orthologs of CLF are found throughout the green lineage, and are the only E(z) homologs in bryophytes, lycopodiophytes, and gymnosperms (Vijayanathan et al. 2022). In *A. thaliana*, CLF is the catalytic subunit of the main PRC2 in the sporophyte and it is required for the proper development of the plant (Baile et al. 2022; Godwin and Farrona 2022). While *A. thaliana clf* mutants display severe phenotypes including dwarfism, CURLY LEAF, and early flowering, the *swn* mutants only show subtle changes during vegetative phase transition (Chanvivattana et al. 2004). To determine the function of BraA.CLF, the only *B. rapa* homolog of CLF (Fig. 1A), we studied the loss-of-function mutant *braA.clf-1*, which exhibited severe developmental alterations, including curved leaves (Fig. 2). While we cannot rule out the presence of secondary mutations in *braA.clf-1*, these phenotypes closely resemble the classic *clf*-mutant phenotypes observed in *A. thaliana*. In addition, we found that *braA.clf-1* displayed early flowering, which is consistent with recent reports indicating that mutations in the Chinese cabbage (*B. rapa ssp. pekkinensis*) *CLF* homolog promotes premature bolting (Huang et al. 2020; Tan et al. 2021). However, the early-flowering phenotype of *braA.clf-1* was not as conspicuous as the Chinese cabbage and *A. thaliana* mutant alleles, due to R-o-18 being already an early-flowering *B. rapa* variety.

The *braA.clf-1* epigenomic profile of H3K27me3 performed in this work (Fig. 4) confirmed the crucial role of BraA.CLF activity in regulating the levels of H3K27m3 in *B. rapa*. However, most of *B. rapa* H3K27me3-marked genes were not hypomethylated in *braA.clf-1,* which is consistent with published genome-wide studies of *clf* alleles in *A. thaliana* (Wang et al. 2016; Carter et al. 2018; Shu et al. 2019). This suggests functional redundancy with other H3K27me3 methyltransferases, likely the *B. rapa* SWN homolog (Fig. 1). In the near future, it will be interesting to explore the function exerted by the other H3K27 methyltransferases in *B. rapa*. On the other hand, our transcriptomic analysis (Fig. 6A) revealed a large number of differentially expressed genes between *braA.clf-1* and the wild-type plants. H3K27me3 is an epigenetic mark associated with gene silencing (Guo et al. 2021). Interestingly, not all the hypomethylated genes were upregulated in *braA.clf-1* (Fig. 7A). These findings are consistent with previous reports indicating that gene expression is not initiated by default in the absence of H3K27me3, but rather requires the coordinated action of multiple chromatin regulators and transcription factors (Schuettengruber et al. 2017; Guo et al. 2021).

Taking advantage of our combined ChIP-seq and RNA–seq analyses, we focused on the set of genes that appeared hypomethylated and upregulated in *braA.clf-1* compared to wild type. These loci very likely include the direct targets of BraA.CLF. The analysis on this dataset showed a significant enrichment on GO terms related to development and gene expression (Supplementary Data S4 and S5). However, we also found a large number of upregulated and downregulated genes related to metabolism in *braA.clf-1* (Supplementary Data S3). These results are consistent with a recent transcriptomic report suggesting that BraA.CLF may be regulating the expression of stress-response and metabolic genes (Nugroho et al. 2023). These authors also proposed that BraA.CLF suppresses the expression of genes involved in the glucosinolate metabolism (Nugroho et al. 2023). However, our analysis only revealed five upregulated genes in *braA.clf-1* related to the glucosinolate-pathway, compared to the 27 genes described by Nugroho and colleagues. Actually, only *BraA03g054450.3C*, a homolog of the *A. thaliana UDP-GLUCOSYL TRANSFERASE 74B1* (*AT1G24100*), and *BraA07g015340.3C* and *BraA07g015330.3C*, two homologs of the *A. thaliana INDOLE GLUCOSINOLATE O-METHYLTRANSFERASE 1* (*AT1G21100*), were upregulated and hypomethylated in *braA.clf-1* leaves. Further research will be needed to define the precise role of BraA.CLF on glucosinolate metabolism.

Our genomic results are consistent with the broad role of CLF and the PRC2 complex previously described in the model plant *A. thaliana* (Shu et al. 2020). For example, as observed in *A. thaliana*, we found that BraA.CLF activity is required to repress the expression of a number of genes related to the floral transition and floral meristem identity. Specifically, the expression of the homologs of floral homeotic genes *AG*, *PISTILLATA* (*PI*), *SEP3* and *SEP4* were strongly upregulated in leaves, likely contributing to the pleiotropic *clf* phenotype. In addition, all the *SOC1* homologs and the main *FT* gene in *B. rapa* were upregulated and showed reduced H3K27me3 levels in *braA.clf-1* leaves (Fig. 7). The upregulation of these floral integrator genes is in agreement with the early-flowering time phenotype of *braA.clf-1*. The *FLC* gene is a well-known target of CLF in *A. thaliana* (Jiang et al. 2008; Lopez-Vernaza et al. 2012). However, no *B. rapa FLC*-*like* gene was misregulated in the *braA.clf-1* mutant. These data confirm that certain floral regulators exhibit differences in gene-expression patterns between *A. thaliana* and *B. rapa* (Calderwood et al. 2021). Further research is necessary to fully understand the complexities of chromatin-dependent regulation of flowering time in Brassica crops.

Our data indicate that BraA.CLF regulates flowering time by affecting the expression of the floral integrator genes *FT* and *SOC1* in *B. rapa*. These observations suggest that, to a certain extent, the methyltransferase activity of CLF is evolutionarily conserved between *A. thaliana* and *B. rapa* following their divergence from a shared ancestor. We believe that the study of the intricate interplay of CLF and other chromatin-modifying factors in crops may offer novel approaches for modulating flowering time to enhance agricultural productivity and sustainability.

### Supplementary Information

Below is the link to the electronic supplementary material.

Supplementary file1 (PDF 314 KB)

Supplementary file2 (PDF 208 KB)

Supplementary file3 (PDF 202 KB)

Supplementary file4 (PDF 267 KB)

Supplementary file5 (DOCX 14 KB)

Supplementary file6 (XLSX 12543 KB)

Supplementary file7 (DOCX 25 KB)

## Data Availability

All ChIP‐seq and RNA‐seq genomic data have been submitted to NCBI Sequence Read Archive under the submission number SUB13676337.
