# Supplementary figures and images for: Brassica rapa CURLY LEAF is a major H3K27 methyltransferase regulating flowering time
Source: Planta. 2024 Jun 12;260(1):27. doi: 10.1007/s00425-024-04454-7 (PMC11169032; doi:10.1007/s00425-024-04454-7)

Figure S1

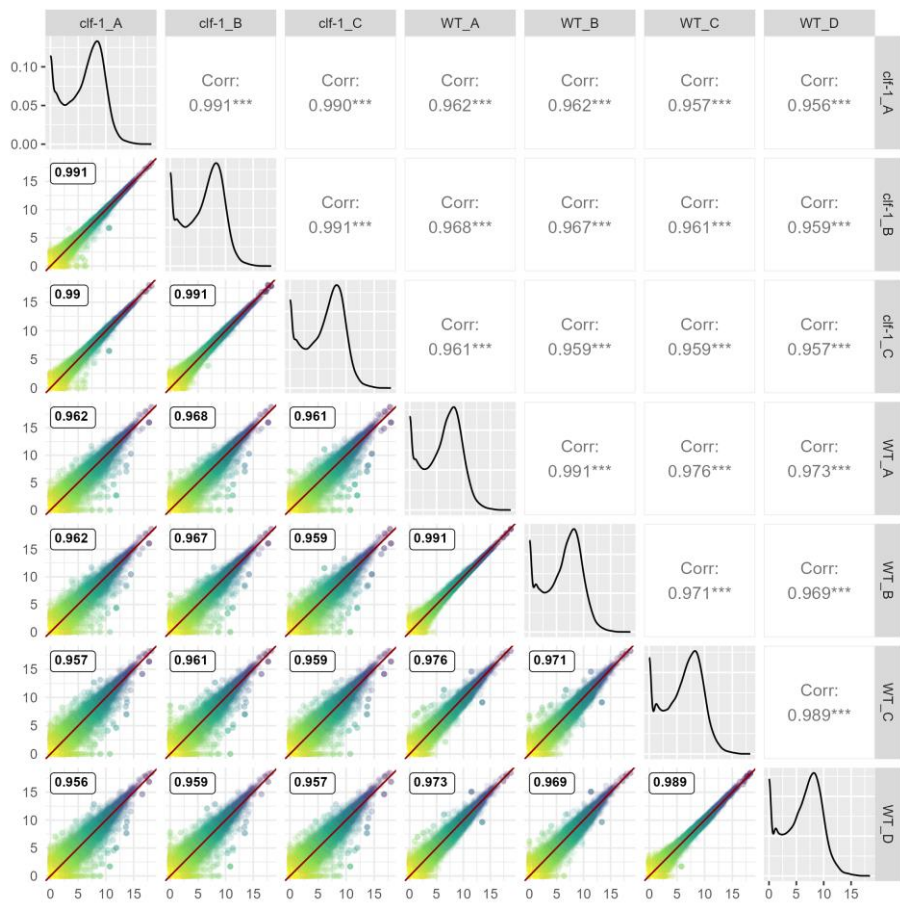

Supplement: Supplementary file 1 — Supplementary file1 (PDF 314 KB) [file 425_2024_4454_MOESM1_ESM.pdf]

Figure S2

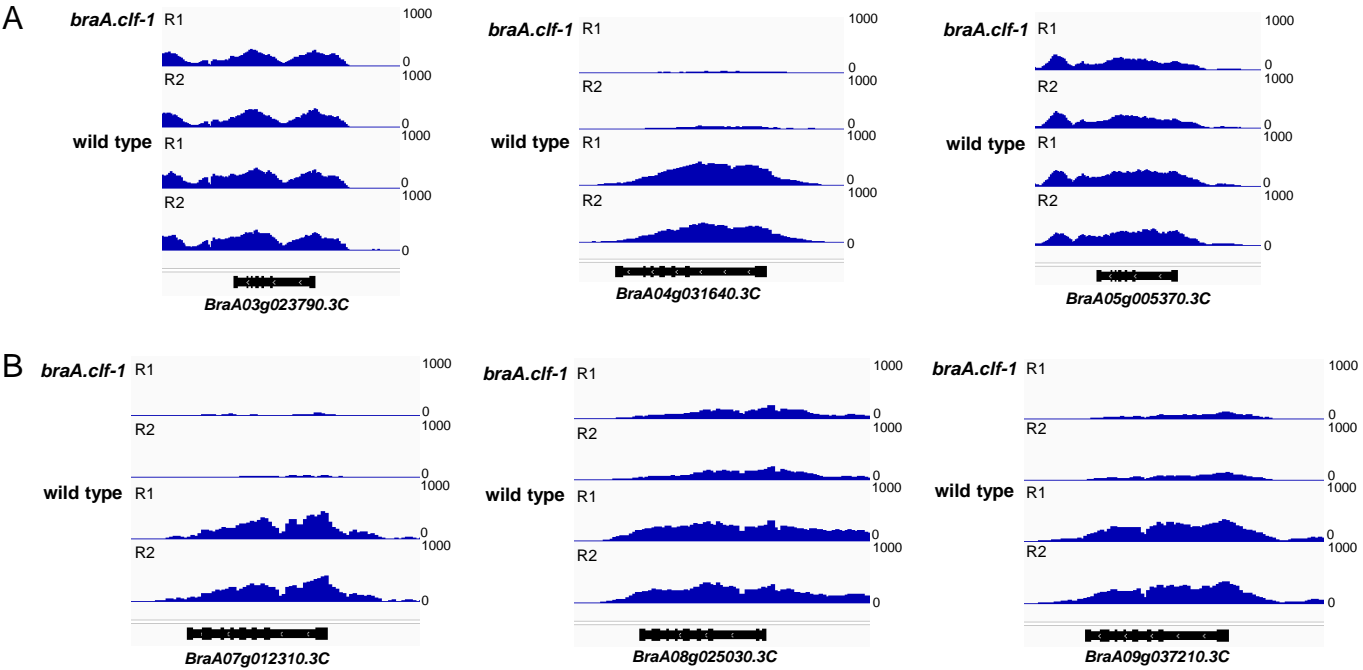

Supplement: Supplementary file 2 — Supplementary file2 (PDF 208 KB) [file 425_2024_4454_MOESM2_ESM.pdf]

Figure S3

BraA.CLF (*BraA04g017190.3C*)

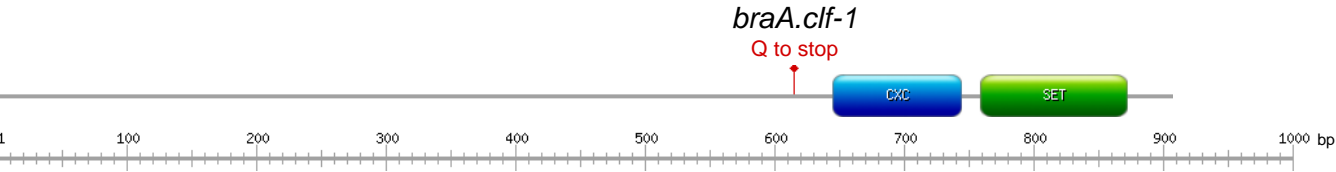

Supplement: Supplementary file 3 — Supplementary file3 (PDF 202 KB) [file 425_2024_4454_MOESM3_ESM.pdf]

Figure S4

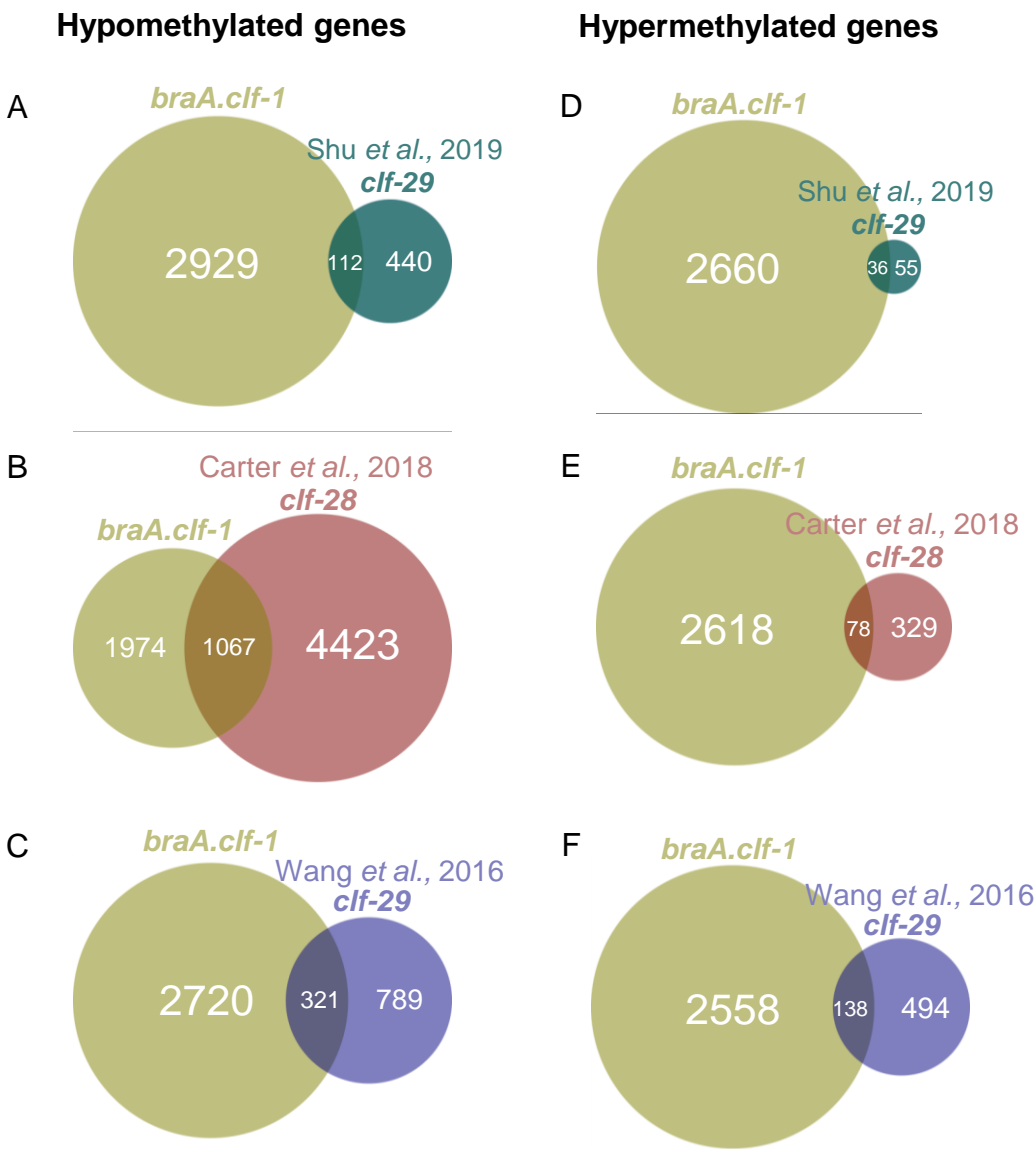

Supplement: Supplementary file 4 — Supplementary file4 (PDF 267 KB) [file 425_2024_4454_MOESM4_ESM.pdf]
