# Supplementary material for: Brassica rapa CURLY LEAF is a major H3K27 methyltransferase regulating flowering time
Source: Planta. 2024 Jun 12;260(1):27. doi: 10.1007/s00425-024-04454-7 (PMC11169032; doi:10.1007/s00425-024-04454-7)
Supplement: Supplementary file 5 — Supplementary file5 (DOCX 14 KB) [file 425_2024_4454_MOESM5_ESM.docx]

**Supplementary Figure legends**

**Fig. S1** RNA-seq sample-sample correlation. Scatter plots showing the pairwise distribution of data points between samples as well as the density plot for each sample and the value of the Spearman correlation. The correlation of gene expression profiles between RNA-seq data from biological replicates was significantly higher compared to the correlation between samples from different genotypes.

**Fig. S2** Integrative Genomics Viewer (IGV) snapshots showing H3K27me3 peaks in *braA.clf-1* and the wild-type in the floral regulatory genes **A** *BraA03g023790.3C*, *BraA04g031640.3C*, and *BraA05g005370.3C*, homologues of *A. thaliana SOC1*; and **B** *BraA07g012310.3C*, *BraA08g025030.3C*, and *BraA09g037210.3C*, homologues of *A. thaliana SEP3*. The two replicates sequenced for the ChIP-seq are shown for each gene (replicate 1 and 2, R1 and R2 respectively).

**Fig. S3** Functional domains of BraA.CLF methyltransferase and location of the *braA.clf-1* mutation. Cartoon showing BraA.CLF protein domain composition. The protein was found to have full identity to a histone lysine N-methyltransferase (PS51576) based on the Prosite domain search tool (<https://prosite.expasy.org/>). BraA.CLF protein contains the evolutionarily conserved SET (PS50280) domain and the preceding CXC (PS51633) domain. The single nucleotide TILLING mutation of *braA.clf-1* mutant plants confers a stop codon (glutamine 615 to stop) upstream the CXC and the catalytic SET domains, presumably abolishing any BraA.CLF histone methyltransferase activity in those plants.

**Fig. S4** Comparison of H3K27me3 hypomethylated and hypermethylated genes in *braA.clf-1* and *A.* *thaliana clf* alleles**. A-C** Venn diagrams showing the overlaps of the H3K27me3 hypomethylated *braA.clf-1* genes determined in this work, and the hypomethylated genes from *A. thaliana* *clf* alelles described in **A** Shu *et al*., 2019, **B** Carter *et al*., 2018, and **C** Wang *et al*., 2016. **D-F** Venn diagrams showing the overlaps of the H3K27me3 hypermethylated *braA.clf-1* genes determined in this work, and the hypermethylated genes from *A. thaliana* *clf* alleles described in **D** Shu *et al*., 2019, **E** Carter *et al*., 2018, and **F** Wang *et al*., 2016. In all cases, the overlap is statistically significant enriched compared to random expectations (hypergeometric test; **(A)** *P* value = 2.92e-16; **(B)** *P* value = 1.96e-156; **(C)** *P* value = 1.05e-83; **(D)** *P* value =8.60e-14 ; **(E)** *P* value =1.32e-08 ; **(F)** *P* value =4.73e-19 ).
