# Supplementary material for: Brassica rapa CURLY LEAF is a major H3K27 methyltransferase regulating flowering time
Source: Planta. 2024 Jun 12;260(1):27. doi: 10.1007/s00425-024-04454-7 (PMC11169032; doi:10.1007/s00425-024-04454-7)
Supplement: Supplementary file 7 — Supplementary file7 (DOCX 25 KB) [file 425_2024_4454_MOESM7_ESM.docx]

**Table S1.** ChIP-seq alignment statistics.

| **Sample** | **Total reads** | **Reads processed^1^** | **Reads mapped** | **Reads duplicated^2^** | **Multi-mapped reads^3^** | **Unmapped reads** |
| --- | --- | --- | --- | --- | --- | --- |
| *Bra.R-o-18_r1** | 54268347 | 54066129 (99.62%) | 42936406 (79.4%) | 6669302 (12.34%) | 7828933 (14.5%) | 11129723 (20.6%) |
| *Bra.R-o-18_r2** | 53797040 | 53598096 (99.63%) | 42049577 (78.5%) | 5896285 (11.00%) | 9488386 (17.7%) | 11548519 (21.5%) |
| *braA.clf-1_r1* | 54489580 | 54186691  (99.444%) | 45007898 (83.1%) | 6259321 (11.55%) | 9264682 (17.1%) | 9178793 (16.9%) |
| *braA.clf-1_r2* | 53942058 | 53676262 (99.57%) | 44526366 (83.0%) | 4904349 (9.14%) | 7885932 (14.7%) | 9149896 (17.0%) |
| INPUT* | 84607814 | 84309011 (99.64%) | 72524708 (86.0%) | 7605807 (9.02%) | 41155708 (48.8%) | 11784303 (14.0%) |

^1^ Removed adapter sequences, bases with quality < 15 and reads with length < 20 bp

^2^ Mapping and optical duplicates were marked

^3^ The highest scoring alignment was kept

* Sequencing data from Poza-Viejo et al. (2022)

**Table S2.** Primers used for RT-qPCR and ChIP-qPCR analyses.

| **Name** | **B. rapa Gene (V3.0)** | **Sequence 5’ - 3’** | **Purpose** | **Tm (ºC)** |
| --- | --- | --- | --- | --- |
| **BraA.FTa -201/-49 F** | *BraA02g016700.3C* | TTCTCGTACATCACTAGACAAGAAAT | ChIP-qPCR | 60 |
| **BraA.FTa -201/-49 R** | *BraA02g016700.3C* | TCGTTCTTGTGAACTAACCCTCT | ChIP-qPCR | 60 |
| **BraA.FTa +21/+165 F** | *BraA02g016700.3C* | TCCTCTTGTGGTAGGGAGAGTT | ChIP-qPCR | 60 |
| **BraA.FTa +21/+165 R** | *BraA02g016700.3C* | CTCAACTCTTGGCTTGTTGAGAAC | ChIP-qPCR | 60 |
| **BraFTa +122/+279 F** | *BraA02g016700.3C* | TGGATCTAAGGCCTTCTCAAGTTC | ChIP-qPCR | 60 |
| **BraFTa +122/+279 R** | *BraA02g016700.3C* | ATGGCCAAGTTATAGTAGAAGACGAA | ChIP-qPCR | 60 |
| **BraA.SOC1 +165/+308 F** | *BraA04g031640.3C* | TGAATTCGCCAGCTCCAAGT | ChIP-qPCR | 58 |
| **BraA.SOC1 +165/+308 R** | *BraA04g031640.3C* | ACACCCTCTCTAAGCAAACG | ChIP-qPCR | 58 |
| **BraA.SOC1 +1045/+1134 F** | *BraA04g031640.3C* | AAACCCCAAATAGACAAGGCT | ChIP-qPCR | 57 |
| **BraA.SOC1 +1045/+1134 R** | *BraA04g031640.3C* | TGGAACTGGAACTCAAAAGCA | ChIP-qPCR | 57 |
| **BraA.SOC1 +2172/+2278 F** | *BraA04g031640.3C* | CCCATGATCGCCATGACCTT | ChIP-qPCR | 60 |
| **BraA.SOC1 +2172/+2278 R** | *BraA04g031640.3C* | TCATGAGATCCCCACTGCAA | ChIP-qPCR | 58 |
| **BraA.FTa F** | *BraA02g016700.3C* | GTTGAGATTGGTGGAGAAGACC | RT-qPCR | 60 |
| **BraA.FTa R** | *BraA02g016700.3C* | ACAATACGAGCACGATACGATG | RT-qPCR | 60 |
| **BraA.SOC1 F** | *BraA04g031640.3C* | GGAGAAAGCTCTAGCTGCAG | RT-qPCR | 60 |
| **BraA.SOC1 R** | *BraA04g031640.3C* | AACATCTAGGTAGGCAACTGTAG | RT-qPCR | 60 |
| **BraA.TUB F** | *BraA10g026070.3C* | CCACTCCTAGCTTTGGTGATCT | RT-qPCR | 59 |
| **BraA.TUB R** | *BraA10g026070.3C* | AGCCACGAGAGGTGAGAGG | RT-qPCR | 60 |

**Table S3.** RNA-seq alignment statistics of *B. rapa.*

| **Sample** | **Total reads** | **Reads processed^1^** | **Reads mapped** | **Multi-mapped reads^2^** | **Unmapped reads** |
| --- | --- | --- | --- | --- | --- |
| *Bra.R-o-18_r1** | 28526252 | 28524567 (99.99%) | 15989027 (56.1%) | 5935499 (20.81%) | 12535540 (43.9%) |
| *Bra.R-o-18_r2** | 23346333 | 23345483 (99.99%) | 13342595 (57.2%) | 4886448 (20.93%) | 10002888 (42.8%) |
| *Bra.R-o-18_r3** | 21715893 | 21715520 (99.99%) | 12235030 (56.3%) | 3933643 (18.11%) | 9480490 (43.7%) |
| *Bra.R-o-18_r4** | 22512828 | 22512448 (99.99%) | 12943010 (57.5%) | 4285173 (19.03%) | 9569438 (42.5%) |
| *braA.clf-1_r1* | 25977445 | 25976886 (99.99%) | 15453707 (59.5%) | 5344150 (20.57%) | 10523179 (40.5%) |
| *braA.clf-1_r2* | 22775974 | 22775428 (99.99%) | 13483769 (59.2%) | 4696827 (20.62%) | 9291659 (40.8%) |
| *braA.clf-1_r3* | 23851763 | 23851273 (99.99%) | 13934654 (58.4%) | 4489935 (18.82%) | 9916619 (41.6%) |

^1^ Removed adapter sequences, bases with quality < 15 and reads with length < 20 bp

^2^ The highest scoring alignment was kept

* Sequencing data from Poza-Viejo et al. (2022)
